# Endothelial dysfunction in preterm infants: The hidden legacy of uteroplacental pathologies

**DOI:** 10.3389/fped.2022.1041919

**Published:** 2022-11-04

**Authors:** Giacomo Simeone Amelio, Livia Provitera, Genny Raffaeli, Matteo Tripodi, Ilaria Amodeo, Silvia Gulden, Valeria Cortesi, Francesca Manzoni, Gaia Cervellini, Andrea Tomaselli, Valentina Pravatà, Felipe Garrido, Eduardo Villamor, Fabio Mosca, Giacomo Cavallaro

**Affiliations:** ^1^ Neonatal Intensive Care Unit, Fondazione IRCCS Ca' Granda Ospedale Maggiore Policlinico, Milan, Italy; ^2^ Department of Clinical Sciences and Community Health, Università Degli Studi di Milano, Milan, Italy; ^3^ Department of Pediatrics, Clínica Universidad de Navarra, Madrid, Spain; ^4^ Department of Pediatrics, Maastricht University Medical Center (MUMC+), School for Oncology and Reproduction (GROW), University of Maastricht, Maastricht, Netherlands

**Keywords:** endothelial dysfunction, endothelium, glycocalyx, preterm infant, preterm birth, chorioamnionitis, dysfunctional placentation, fetal growth restriction

## Abstract

Millions of infants are born prematurely every year worldwide. Prematurity, particularly at lower gestational ages, is associated with high mortality and morbidity and is a significant global health burden. Pregnancy complications and preterm birth syndrome strongly impact neonatal clinical phenotypes and outcomes. The vascular endothelium is a pivotal regulator of fetal growth and development. In recent years, the key role of uteroplacental pathologies impairing endothelial homeostasis is emerging. Conditions leading to very and extremely preterm birth can be classified into two main pathophysiological patterns or endotypes: infection/inflammation and dysfunctional placentation. The first is frequently related to chorioamnionitis, whereas the second is commonly associated with hypertensive disorders of pregnancy and fetal growth restriction. The nature, timing, and extent of prenatal noxa may alter fetal and neonatal endothelial phenotype and functions. Changes in the luminal surface, oxidative stress, growth factors imbalance, and dysregulation of permeability and vascular tone are the leading causes of endothelial dysfunction in preterm infants. However, the available evidence regarding endothelial physiology and damage is limited in neonates compared to adults. Herein, we discuss the current knowledge on endothelial dysfunction in the infectious/inflammatory and dysfunctional placentation endotypes of prematurity, summarizing their molecular features, available biomarkers, and clinical impact. Furthermore, knowledge gaps, shadows, and future research perspectives are highlighted.

## Prematurity: endotypes and endothelium

Preterm birth is defined as birth prior to 37 weeks of gestational age (GA). Prematurity, particularly in the lowest gestational age ranges, is the leading cause of infant mortality and morbidity, accounting for up to 35% of all deaths among newborns, and also may lead to severe complications as well as prolonged stays in neonatal intensive care units (NICUs) (1–3). Although considerable progress has been made in identifying factors that contribute to preterm birth and its complications, several gaps remain in our understanding of the pathogenesis, pathology, and clinical management of the condition. Preterm delivery is induced by the interaction of various medical and genetic causes but also by environmental and socioeconomic determinants (4, 5). In recent years, two broad pathophysiological patterns have been identified as responsible for the majority of very and extremely preterm births (i.e., GA less than 32 weeks): intrauterine infection/inflammation (Table 1) and dysfunctional placentation (Table 2) (10). These patterns involve specific cellular and biochemical pathways, representing two distinct *endotypes* of prematurity (11, 12).

**Table 1:** Infectious/inflammatory endotype.

Classification and epidemiology	
Conditions	• Preterm labor • PROM • Cervical insufficiency • Placental abruption^a^
Physiopathology	FIRS with cytokine storm and leukocyte activation, frequently associated with infectious placental pathology
Placental hallmarks	Histological corioamnionitis
Timing of trigger	Acute
Delivery	Mostly spontaneous onset of labor
Gestational Age (mean)^b^	<28 weeks, decreasing frequency with advancing GA
Birth weight percentile	Low, normal, or high
**Endothelial dysfunction and clinical implications**	
Endothelial features	Luminal endothelial surface transformations: glycocalyx shedding, adhesion molecules exposure, activation and consumption of platelets and coagulation factors, dysregulated vascular permeability, including the blood-brain barrier
Endothelial biomarkers	• Syndecan-1, Heparan sulfate, Hyaluronan, • P-selectin, E-selectin, ICAM-1, VCAM-1, • Ang-1, Ang-2 • TF
Alarming cardiovascular implications	Myocardial dysfunction, systemic hypotension, tachycardia, distributive shock in severe sepsis
Endothelium-associated complications	Diffused tissue edema, brain white matter injury, hemostatic incompetence

PROM, premature rupture of membranes; FIRS, fetal inflammatory response syndrome; GA, gestational age; Ang, angiopoietin; TF, tissue factor.

^a^ Although a 9-fold higher risk in CA and PROM, placental vascular abnormalities and severe hypertensive disorders are other established risk factors for abruption, requiring a heedful anamnestic evaluation to classify the endotype properly (6, 7).

^b^ Considering cohorts of very preterm infants (8, 9).

**Table 2:** Dysfunctional placentation endotype.

Classification and Epidemiology	
Conditions	• Preeclampsia • Fetal growth restriction
Physiopathology	Reduced trophoblast invasion with feto-placental underperfusion
Placental hallmarks	Vascular underperfusion lesions
Timing of trigger	Chronic
Delivery	Mostly medically indicated
Gestational Age (mean)^a^	>28 weeks, increasing frequency with advancing GA
Birth weight percentile	Low
**Endothelial dysfunction and Clinical Implications**	
Endothelial features	Malperfusion, oxidative stress, imbalance of angiogenic and vasomotor factors leading to fetal developmental reprogramming with long-term outcomes
Endothelial biomarkers	• H_2_O_2_, malondialdehyde, protein carbonyl groups, glutathione peroxidase • sFlt-1, sEng, VEGF, PlGF, IGF-1 • ADMA, Nitrate, Nitrite, CO, H2S, ET-1
Alarming cardiovascular implications	Myocardial remodeling and hypertrophy, transient systemic hypotension, persistent pulmonary hypertension, vascular stiffness
Endothelium-associated complications	BPD-associated pulmonary hypertension, ROP, metabolic syndrome in adulthood

GA, gestational age; H_2_O_2_, hydrogen peroxide; sFlt-1, soluble fms-like tyrosine kinase-1; sEng, soluble endoglin; VEGF, vascular endothelial growth factor; PlGF, placental growth factor; IGF-1, ADMA, asymmetric dimethylarginine; CO, carbon monoxide; H_2_S, hydrogen sulfide; ET-1, endothelin-1; BPD, bronchopulmonary dysplasia; ROP, retinopathy of prematurity.

^a^ Considering cohorts of very preterm infants (8, 9).

The term endotype refers to “a subtype of a condition, which is defined by a distinct functional or pathophysiological mechanism” (13). Endotypes are thus a different form of classification from clinical phenotypes and describe distinct disease entities with a defining etiology and/or a consistent pathophysiological mechanism (13, 14). Although the term endotype has been applied mainly to studies of respiratory and allergic diseases, its use is increasingly extended to other areas of medicine (14–19). In line with the approach of personalized medicine, endotyping will facilitate the design of more targeted therapeutic and prognostic approaches (14–17, 19). Various epidemiological studies have suggested a correlation between endotypes of prematurity and neonatal phenotypes, although much remains to be explained about how antenatal events determine the outcomes (8, 9, 11, 20).

The vascular endothelium (VE) is a monolayer of cells covering the luminal surface of blood vessels (21). The VE plays an essential role in vascular homeostasis and regulates several pathways that could reveal information about the neonatal endotype-phenotype correlation (22). In particular, endothelial cells and pericytes interact with soluble mediators of inflammation, modulate vascular permeability and coagulation balance, drive angiogenesis, and regulate vascular tone through continuous endocrine-paracrine activity (23, 24). Conversely, VE undergoes changes in its properties when exposed to injuries, shifting towards a proinflammatory, procoagulative, and vasoconstrictive phenotype, resulting in the condition known as endothelial dysfunction (25).

Over the past two decades, endothelial pathways have proved to be an effective diagnostic and therapeutic target in several adult inflammatory diseases, increasing interest in the role played by VE in many medical areas, including prematurity-related complications. To date, examples of this research are already available, such as antiangiogenetic therapy with anti-Vascular Endothelial Growth Factor (VEGF) drugs for retinopathy of prematurity (ROP) (26). Moreover, several studies documented endothelial dysfunction in former premature adults, with increased incidence of high blood pressure, type 2 diabetes, and stroke, suggesting that prematurity may be the origin of an unfavorable endothelial phenotype (20, 27–29). Interestingly, the placental and pregnancy studies revealed that this endothelial dysfunction may have started very early and be related to the adverse intrauterine environment induced by the pregnancy complications that lead to preterm birth (30, 31).

Gaps in knowledge about endothelial dysfunction in preterm infants limit progress on prevention, early diagnosis, and treatment of the condition. Furthermore, characterizing endothelial profiles in fetuses and preterm infants is highly challenging due to the “endothelial developmental physiology” process (32–34). Several factors, such as GA, partial arterial oxygen pressure (PaO2), and placental vascular growth factors, expose the immature VE to continuous dynamic events during the antenatal period. Additionally, simultaneous maturation of the hemostatic and immune systems, closely connected to the VE, adds additional complexity to understanding the process (35, 36). Thus, we should not consider fetal endothelial physiology as static but consider it a developmental process that continues in postnatal life and progressively approaches the adult phenotype.

Classifying prematurity into endotypes can provide a systematic model for approaching the physiopathology of “endothelial phenotypes” commonly encountered in NICU, such as systemic hypotension, pulmonary hypertension, generalized tissue edema, and thrombotic or hemorrhagic events.

Rather than surveying all known mechanisms of endothelial damage in prematurity, this review focuses on the main endothelial features of the infectious/inflammatory and dysfunctional placentation endotypes, highlighting their peculiar pathways and clinical implications in the neonatal setting.

## Infectious/inflammatory endotype

### Classification and pathogenesis

The infectious/inflammatory endotype is associated with preterm labor, premature rupture of membranes (PROM) out of labor, cervical insufficiency, and placental abruption (10, 37, 38) (Table 1). These conditions, even though distinct, are frequently associated with chorioamnionitis, defined as an acute inflammation of the membranes and chorion of the placenta, typically caused by an ascending microbial infection (39, 40). Of note, placental abruption can also be associated with placental vascular abnormalities and severe hypertensive disorders, requiring a heedful anamnestic evaluation to classify the endotype properly (6, 7).

The incidence of chorioamnionitis becomes higher as GA decreases and can be as high as 90% in infants at 24 weeks of GA and then progressively decreases to 10% in late preterm infants (GA > 34 weeks) (41). In addition, a growing number of individual studies and meta-analyses show an association between chorioamnionitis and increased risk of developing complications of prematurity such as necrotizing enterocolitis (NEC), early- and late-onset sepsis, periventricular leukomalacia, bronchopulmonary dysplasia (BPD), ROP, intraventricular hemorrhage, or patent ductus arteriosus (8, 42–51). However, a common conundrum in perinatal medicine is the extent to which chorioamnionitis, as well as other pregnancy complications, harms preterm infants through triggering prematurity or through disturbances in fetal homeostasis and development.

During chorioamnionitis, the infection invades the decidua and the amniotic cavity leading to neutrophilic infiltration of the chorioamnion and causing an increase in proinflammatory cytokine concentrations in the amniotic fluid (52, 53). As inflammation progresses, immune cells can penetrate blood vessels and infiltrate the umbilical cord, resulting in funisitis, which reflects a systemic inflammatory response syndrome called Fetal Inflammatory Response Syndrome (FIRS) (54).

The term FIRS was initially coined in 1998 to identify an elevated concentration of fetal plasma interleukin (IL)-6 in response to an intra-amniotic infection in spontaneous preterm labor (54). However, the type, kinetics, and effects of its numerous proinflammatory markers were characterized only subsequently and still remain incomplete. Moreover, due to practical and ethical limitations, most of the information available derives from preclinical studies (30).

**Figure 1:**
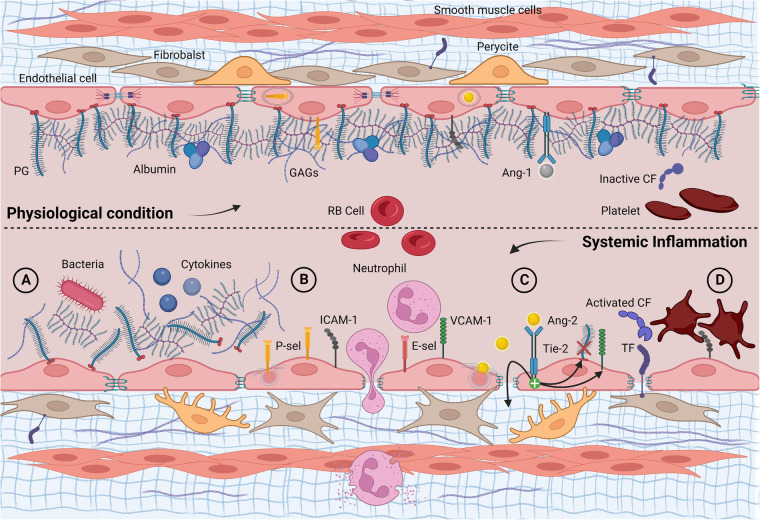
Longitudinal section of a blood vessel: endothelial phenotype in physiological conditions and infectious/inflammatory endotype. (**A**) Acute glycocalyx shedding following endogenous and/or exogenous proinflammatory stimuli; (**B**) unveiling and overexpression of membrane glycoproteins, adhesion, and diapedesis of neutrophils; (**C**) Ang-2 exocytosis with increased vascular leakage, further glycocalyx shedding *via* heparanase and up-regulation of adhesion molecules; (**D**) subendothelial TF exposure, activation, and consumption of clotting factors and platelets. Ang-1, angiopoietin 1; Ang-2, angiopoietin 2; CF, clotting factor; E-sel, E-selectin; GAGs, glycosaminoglycans; PG, proteoglycan; P-sel, P-selectin; RB cell, red blood cell; TF, tissue factor; Tie-2, tyrosine kinase receptor. Created with BioRender.com.

The incidence of FIRS, likewise for histological chorioamnionitis, is inversely related to GA, and its extent is proportional to the maturity of the immune system (55). In this context, premature infants exposed to an infectious/inflammatory endotype may manifest a broad spectrum of endothelial dysfunction phenotypes. However, the FIRS model is the condition that best characterizes the endothelial response of the fetus and preterm infant to this group of adverse antenatal conditions. Therefore, in the following paragraphs, we will summarize the endothelial pathways with an increased risk of being affected by a systemic inflammatory response syndrome in the setting of an infectious/inflammatory endotype of prematurity (Figure 1, Table 1).

### Role of the endothelial glycocalyx

#### Molecular basis

Communication between the bloodstream and endothelial surface plays a crucial role during systemic inflammation. Under physiological conditions, this interaction is mediated by the endothelial glycocalyx, an antiadhesive and anticoagulant layer that covers the luminal endothelial surface (56). The endothelial glycocalyx is mainly composed of proteoglycans, macromolecules with a protein core anchored to the endothelial surface or released soluble in the extracellular compartment (57). A large amount of glycosaminoglycan, mostly heparan sulfate and hyaluronan, gives the endothelial glycocalyx great hydrophilicity, which allows for trapping a fixed noncirculating plasma volume of approximately one liter in adults (58). The result is a soluble gel-like network, whose geometry changes depending on the vascular bed, volume load, and blood flow rates, and that acts as a mechanotransducer, translating shear stress into cellular signaling processes (59). Moreover, the negative charge of glycosaminoglycans repels endothelial glycocalyx interaction with negatively charged proteins as well as white and red blood cells and platelets, maintaining an oncotic gradient that limits vascular leakage (60). Conversely, entrapped plasma- and endothelium-derived proteins help in reducing hydraulic conductivity, maintaining an anticoagulant phenotype, and guaranteeing cell signaling (61).

In addition, glycoproteins are essential for leukocyte recruitment, platelet activation, and hemostatic function (59). The endothelial glycocalyx coating provides a physical barrier that prevents the indiscriminate activation of all these pathways. However, it undergoes significant changes in acute inflammation, especially when exposed to lipopolysaccharides (LPS) (62). Following neutrophilic activation, the release of reactive oxygen species (ROS), reactive nitrogen species (RNS), and tumor necrosis factor-α (TNF-α) lead to glycocalyx degradation *via* heparanase (62, 63). This process promotes endothelial glycocalyx shedding and severely affects its functions (64).

#### Perinatal evidence and perspectives

Experimental models showed that endothelial glycocalyx is expressed early in ontogenesis for normal vasculogenesis and angiogenesis (65, 66). Many proteins constituting the glycosaminoglycans are expressed even before the onset of vascular circulation (67). Furthermore, during embryological development, endothelial glycocalyx appears from the very first moment blood flow emerges, and the glycosaminoglycan structure shows a regular composition similar to the adult in a quail embryo model (68). A human *in vivo* study investigated endothelial glycocalyx thickness by testing the perfused boundary region, an inverse measure of glycocalyx thickness, in a population of full-term and preterm infants (69). Interestingly, premature neonates in the lowest GA group had the largest endothelial glycocalyx dimensions at birth, which highlights the relevance of endothelial glycocalyx in the development of the circulatory system (69).

All inflammation conditions, such as sepsis, autoinflammatory diseases, surgery, and trauma, can cause endothelial glycocalyx damage (60, 70). Although the evidence is not robust, this process may also occur in preterm infants. At follow-up, the cohort of neonates in the lowest GA group showed a progressive thinning of the glycocalyx, expressed as an increase of the perfused boundary region, reaching lower glycocalyx dimensions compared to infants of higher GA. This suggests that peri- and postnatal inflammatory insults may damage glycocalyx integrity (69). Moreover, degradation of the endothelial glycocalyx layer (shedding) has also been demonstrated in a cohort of neonates undergoing cardiac surgery (71). Interestingly, high preoperative doses of corticosteroids seem to confer protection to the endothelial glycocalyx through unresolved mechanisms, opening the hypothesis of benefits on endothelial glycocalyx induced by antenatal steroids (72, 73).

Syndecan-1 (an anchored protein core), heparan sulfate, and hyaluronan are the three well-established biomarkers of endothelial glycocalyx disruption (60, 70). The products of glycosaminoglycan chains increased in the plasma and urine of patients during and after the inflammatory event, generating a further amplification of the inflammatory cascade. Vascular loss of syndecan-1 resulted in reduced expression of shear stress compensating pathways, such as nitric oxide (NO) production (74). Moreover, hyaluronan fragments activate nuclear factor-κβ (NF-κβ) signaling, which produces proinflammatory cytokines and chemokines. At the same time, serum heparan sulfate induces myocardial dysfunction, which plays a crucial role in systemic hypotension and organ hypoperfusion during neonatal septic shock (64, 75, 76).

Clinical studies targeting the perinatal role of the endothelial glycocalyx in the infectious/inflammatory endotype may have great diagnostic and therapeutic perspectives for patient-tailored care. The endothelial glycocalyx breakdown could be crucial in septic preterms and infants with non-infectious hemodynamic imbalances (76). Unstable hemodynamic conditions are expected in very preterm infants in the first days of life and are usually treated with high intravenous volume administration and inotropes (77). Nevertheless, fluid overload may promote adverse outcomes through endothelial glycocalyx disruption, worsening of myocardial dysfunction, vascular leak, systemic inflammatory cascade, and organ damage (60). Likewise, excessive use of vasoactive amines has a potentially harmful effect on endothelial glycocalyx (78).

Interestingly, preclinical data showed that colloids favor endothelial glycocalyx restoration (79–83). Therefore, besides volume, the choice of fluid therapy could also affect the endothelial phenotype (79). Indeed, albumin is a physiological component of endothelial glycocalyx and possesses pleiotropic physiological activities, including antioxidant effects and protective effects on vessel wall integrity (84). However, so far, there are no supporting trials for albumin first-line use in this clinical setting (85). In conclusion, targeted therapies that preserve endothelial glycocalyx shedding may potentially have protective cardiovascular effects. However, these clinical applications still need a long way to go.

### Adhesion molecules unveiling and overexpression

#### Molecular basis

As a result of damage to the endothelial glycocalyx, adhesion molecules are exposed to the denuded VE, which induces the recruitment of leukocytes and platelets, promoting endothelial dysfunction (86, 87). The endothelial glycoproteins responsible for leukocyte adhesion mainly belong to the selectins family (P- and E-selectin) and the endothelial immunoglobulin superfamily, such as the intercellular adhesion molecule 1 (ICAM-1) and the vascular cell adhesion molecule 1 (VCAM- 1). Their constitutive expression varies significantly between tissues, and their kinetic upregulation is diversified (88). Especially, ICAM-1 has a broad physiological expression in most vascular beds, P-selectin is stored and rapidly mobilized by Weibel-Palade bodies, while E-selectin and VCAM-1 have a low constitutive expression, rising mainly during inflammatory stimulus *via* nuclear transcription (89–91).

The complex cascade mechanisms triggered by adhesion molecules are finely regulated (89, 91, 92). Briefly, initial adhesive interactions between leukocytes and venular endothelium are carried out by P- and E-selectins *via* low-affinity interactions that lead to leukocyte tethering and rolling on the endothelial surface. Subsequently, leukocyte activation reinforces this interaction, exposing leukocyte integrins and binding the endothelial immunoglobulin superfamily (89). Then, having reached a stable adhesion, platelet endothelial cell adhesion molecule-1 (PECAM-1) and other endothelial immunoglobulins permit leukocyte diapedesis through the intercellular spaces (93).

The systemic inflammatory response, including FIRS, intensifies this process by overexpressing adhesion molecules. The mechanism involves several molecular pathways: cytokines, endotoxins, shear stress, ROS, RNS, and imbalance of glucose and cholesterol are the main biological regulators (90).

#### Perinatal evidence and perspectives

Despite some differences due to a reduced baseline exposure of glycoproteins and delayed recruitment of neutrophils in tissue, evidence suggests that the adhesion molecules of preterm infants are not anergic to proinflammatory stimuli (76). *Ex vivo* studies demonstrated that human umbilical vein endothelial cells (HUVECs) of extremely low birth weight infants (ELBW) could upregulate E-selectin, VCAM-1, and ICAM-1 in response to TNF-α and IL-1β (94). This setting is reliable as the fetal sheep undergoes cytokine changes as early as 5 h after the intraamniotic injection of LPS and up to 15 days after the onset of inflammation (95). Furthermore, the comparative study of neonatal and adult selectins showed effective functionality *in vitro*, with no differences in rolling speed or number of adherent cells (96). However, *in vivo* characterization of adhesion molecules on premature neonates has been scarcely investigated.

The cord blood sample of ELBW infants showed enhanced exposure and shedding of soluble E-selectin and ICAM-1 in patients with funisitis, demonstrating that chorioamnionitis with funisitis induces a significant glycoprotein overexpression with soluble forms increased in the fetal circulation (97).

The transient vascular shedding of glycoprotein suggested using soluble adhesion molecules as a surrogate marker to assess the endothelial inflammatory response severity. Several studies on term infants with sepsis exploited this process, finding greater sensitivity and predictivity than C-reactive protein alone (98–100). However, the clinical use of these markers in preterm infants is limited by physiological fetal immaturity and a lack of normality curves adapted for GA (96, 101, 102). This mainly concerns selectins, whose transcription factor, NF-κβ has a GA-dependent capacity (94). Amelio et al. supported these findings by showing lower plasma E-selectin levels in preterm infants compared to a control group of healthy full-term infants, despite having a predominantly inflammatory endotype with higher VCAM-1 level (103). Therefore, the clinical application of these markers requires further research.

### Angiopoietins imbalance and vascular leakage

#### Molecular basis

Emerging evidence points toward a “double barrier concept” in which both the endothelial cell layer with its multiple intercellular junctions and endothelial glycocalyx play a role in maintaining the vascular barrier (104). Although both layers must be compromised to obtain a major increase in vascular permeability, a slight increase in cell permeability in a damaged endothelial glycocalyx may be sufficient to generate vascular leakage (105). In addition, paracellular and transcellular pathways are linked to water and macromolecule leakage from the endothelial cell layer (106). The first is regulated by endothelial cleft, with ROS and TNF-α stimulating the opening of tight, adherent, and gap junctions (107, 108). The second is controlled by proinflammatory mediators known to accelerate caveolae formation, leading to trans-endothelial hyperpermeability (109). Several growth factors and receptor systems are involved in these processes. However, the angiopoietin (Ang)/tyrosine kinase receptor with immunoglobulin and epidermal growth factor homology domain 2 (Tie-2) signaling pathway seems particularly relevant.

The angiopoietin family (Ang-1, Ang-2, Ang-3, Ang-4) was initially discovered as a regulator of angiogenesis and subsequently demonstrated a key role in several inflammatory diseases through modulation of the Tie-2 receptor (22). Ang-1 is a potent stabilizer of endothelial cell contacts, modulating the VEGF/NO pathway (110). It is constitutively expressed by pericytes, smooth muscle cells, and fibroblasts and promotes endothelial quiescence by enhancing cell survival and downregulating proinflammatory and pro-coagulating pathways (111). In addition, Ang-1 has cardiovascular protective effects, showing a therapeutic impact in the post-ischemic myocardium by improving cardiac function, scar thickness, and scar area fraction (112).

Ang-2 is a competitive inhibitor of Ang-1. It is undetectable in quiescent VE but increases dramatically *via* rapid exosomal secretion during hypoxic or inflammatory injury. Assisted by VEGF, Ang-2 promotes destabilization of both layers of the vascular barrier: it reduces endothelial glycocalyx thickness by activating heparanase and increasing soluble heparan sulfate. Moreover, Ang-2 subverts intercellular junctions inducing paracellular gap formation and increasing the surface expression of cell adhesion molecules, worsening edema and tissue neutrophil recruitment (105, 113). All these properties indicate Ang-2 as an important mediator of LPS-induced endothelial glycocalyx damage (105, 114). Ang-3 in mice and Ang-4 in humans are interspecies orthologs (115). They are both Tie-2 agonists, although they are still poorly characterized (116).

#### Perinatal evidence and perspectives

Plasma imbalance of the Ang-2/Ang-1 ratio has been demonstrated in acute lung injury, sepsis, trauma, and multi-organ dysfunction in full-term neonates, children, and adults, also exhibiting a predictive value on mortality (117–120).

Ang/Tie-2 pathway is directly involved in the infectious/inflammatory endotype of prematurity. A placental immunohistochemical study demonstrated a significant increase in Tie-2 angiopoietin receptors in chorioamnionitis and funisitis (121). Furthermore, a higher concentration of Ang-2 was found in the amniotic fluid of women with preterm labor and intra-amniotic inflammation compared to women with preterm birth without inflammation (122). A plasma imbalance towards Ang-2 was shown from the first hours of life in a cohort of very preterm infants, suggesting an antenatal origin of this endothelial phenotype (103). Moreover, Ang-2 concentrations were inversely related to GA, suggesting a co-dependence between prematurity and endothelial permeability (103, 122).

Finally, the Ang/Tie-2 system may play a role in the close correlation between the infectious/inflammatory endotype and adverse neurological outcomes. During FIRS, the impaired integrity of the blood-brain barrier (BBB) increases the permeability to proinflammatory cells and cytokines, which causes a direct injury to neurons and oligodendrocytes of the developing brain (123). Preclinical models show that Ang-2 increases BBB permeability *via* paracellular and transcellular routes (124). Furthermore, Ang-2 levels were upregulated in patients affected by cerebrovascular disorders associated with BBB alterations (124). However, to our knowledge, there are still no studies on the correlation between Ang-2 and neurological outcomes in preterms.

### Hemostatic changes

Hemostatic changes in infants with an infectious/inflammatory endotype also deserve attention. Besides leukocyte diapedesis and vascular leakage, vascular surface mutation activates platelets and coagulation. Moreover, inflammation downregulates anticoagulant proteins and inhibits the fibrinolytic system, according to the immunothrombosis process (125, 126). On the other hand, in advanced septic states, an indiscriminate activation leads to the consumption of hemostatic factors, resulting in disseminated intravascular coagulation (DIC) and subsequent hypocoagulability (127). However, the evidence for a hyper- or hypocoagulant endothelial phenotype during FIRS is currently scarce.

Despite expressing different concentrations of pro- and anticoagulant factors, premature infants are hemostatically competent, and viscoelastic tests of infants with systemic inflammation have shown significant but not univocal alterations of the hemostatic balance (128–136). In addition, recent studies have observed a lengthening of coagulation times during neonatal sepsis and post-surgery, so the bleeding risk in these newborns must be considered (130). However, as in adults, an opposite prothrombotic switch cannot be excluded in the initial phase of the infectious/inflammatory endotype (137).

In this regard, the high levels of IL-1β observed in FIRS showed procoagulant and permeabilizing effects on HUVECs through a rapid and dose-dependent increase in tissue factor activity (138). Similarly, HPA increased coagulation activity *via* the stimulation of tissue factor expression in endothelial cells (139). Therefore, exploring the tissue factor pathway could bridge coagulation, angiopoietins, and endothelial glycocalyx shedding in the perinatal period (103). Further viscoelastic studies will help to better characterize the perinatal hemostatic peculiarities related to this endotype.

## Dysfunctional placentation endotype

### Classification and pathogenesis

The placenta is the maternal-fetal interface, and its vascular development is crucial for maintaining proper fetal growth through efficiently delivering nutrients and oxygen (140). The unique characteristics of the placental and umbilical cord vascular endothelium provide information on the events occurring on both sides. Therefore, several studies have investigated the physiology and diseases of placentogenesis, providing helpful information on the aberrant pathways leading to several disorders of pregnancy (141, 142).

The dysfunctional placentation endotype mainly encloses hypertensive disorders of pregnancy, including gestational hypertension, preeclampsia, and eclampsia, as well as fetal growth restriction (FGR), defined as the failure of a fetus to achieve its genetic growth potential (10, 141).

Severe dysfunctional placentation endotype may require the earlier birth of the infant, balancing the neonatal risks of prematurity with the fetal risk of carrying on a pregnancy burdened by pathology.

These conditions are among the most significant contributing factor to perinatal morbidity and mortality, affecting up to 12%–16% of pregnant patients (143, 144). Furthermore, FGR is the leading risk factor for stillbirth and may increase the risk of developing several complications of prematurity, including BPD and ROP (9, 145–148).

Preeclampsia and FGR are often associated and share similar pathogenesis, such as reduced trophoblast invasion and impaired perfusion in the uteroplacental compartment, leading to harmful effects to the fetus (149, 150). Usually, the expression of integrins, cadherins, and metalloproteases allows the cytotrophoblasts to penetrate the uterine wall between the 6th and 8th week of gestation, differentiating into an endothelial phenotype and transforming the spiral arteries into large vessels with low vascular resistance (140). These steps increase the blood flow directed to the intervillous space and the fetus during early development. Conversely, failure of this process leads to narrow, high-strength, high-pressure spiral arteries, resulting in chronic placental hypoperfusion (151). The hallmarks of this impaired uteroplacental blood flow are maternal vascular underperfusion lesions on placental histology (152).

The mismatch between fetoplacental blood demand and adequate uteroplacental supply promotes blood flow turbulence, hyperinflammatory state, and placental endothelial dysfunction (151, 153, 154). An increase in maternal inflammatory cytokines, acute phase proteins, and systemic endothelitis is well documented in severe preeclampsia, mainly when associated with FGR (155, 156). On the fetal side, the consequence is a preferential blood flow redistribution to the vital organs (brain, myocardium, and adrenal glands) at the expense of other organs (157). Furthermore, the high placental resistance associated with FGR may lead to increased cardiac afterload, resulting in myocardial remodeling and hypertrophy, increased vascular tone, vascular stiffness, and risk of perinatal hypotension and persistent pulmonary hypertension (157).

**Figure 2:**
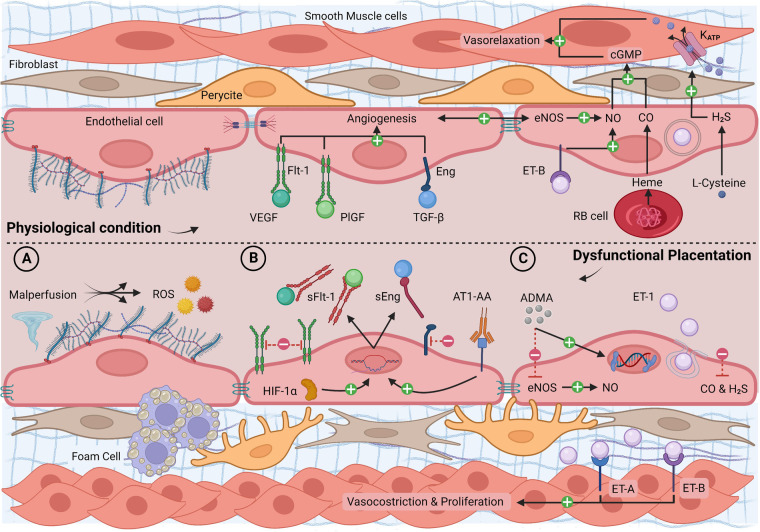
Longitudinal section of a blood vessel: endothelial phenotype in physiological conditions and dysfunctional placentation endotype. (**A**) Malperfusion phenomena with shear stress, hypoxia, reperfusion injury, atherosclerosis, and ROS production; (**B**) high levels of HIF-1α and AT1-AA with increased synthesis of sFlt-1 and sEng, reduced free circulating VEGF, PlGF, TGF-β, and reduced angiogenesis; (**C**) high levels of ADMA and ET-1 and low levels of gasotransmitters with increased vasoconstriction, smooth muscle layer proliferation, and epigenetic mutations. ADMA, asymmetric dimethylarginine; AT1-AA, angiotensin II type 1 agonistic receptor autoantibody; cGMP, cyclic guanosine monophosphate; CO, carbon monoxide; eNOS, endothelial nitric oxide synthase; ET-1, endothelin-1; ET-A/B, endothelin-1 receptor A/B; H_2_S, hydrogen sulfide; HIF-1α, hypoxia-inducible factor 1α; Katp, potassium channel; PlGF, placental growth factor; RB cell, red blood cell; ROS, reactive oxygen species; sEng, soluble endoglin; sFlt-1, soluble fms-like tyrosine kinase-1; TGF-β, transforming growth factor-β; NO, nitric oxide; VEGF, vascular endothelial growth factor. Created with BioRender.com.

Despite overlaps with previously described inflammatory pathways, the different triggers and chronic exposure of this endotype lead to typical molecular and phenotypic features that are reviewed below and summarized in Figure 2 and Table 2.

### Perfusion mismatch and oxidative distress

#### Molecular basis

Deficient spiral arterial remodeling leads to high velocity and pulsatile inflow in the intervillous space, greater intermittent perfusion of the placenta, atherotic changes with vascular wall deposition of foam cells, platelet aggregation, and fibrinoid necrosis (150). In addition, impaired perfusion induces hypoxia/reperfusion injury with increased oxidative stress (158).

Highly reactive free radicals can negatively affect lipids, proteins, or nucleic acids (158). ROS and RNS play an important physiological role at normal levels, acting as second messengers in many signaling pathways (159, 160). However, cell damage and cell death are triggered when ROS and RNS production overwhelms antioxidant capacity (158, 161–168).

Pregnancy represents, *per se*, a state of oxidative stress resulting from the increased metabolic activity in the placental mitochondria and the reduced scavenging power of antioxidants but this imbalance increases as a consequence of placental dysfunction (169–171). Indeed, free radical species inevitably rise with cascade effects on inflammation, angiogenesis, and vascular contractility. The overexpression of free radicals and acute phase proteins is an established process in pregnancies complicated by preeclampsia or FGR, involving even severe cardiovascular effects (172, 173).

#### Perinatal evidence and perspectives

Several studies investigated, through cord blood samples analysis, the oxidant and antioxidant state of newborns of preeclamptic women, with or without FGR, compared to newborns from physiological pregnancies (156, 174–183). Different analytical methods and some biomarkers have been used to measure oxidative stress, such as direct detection of ROS (e.g., hydrogen peroxide, H_2_O_2_), indirect research of the peroxidation products of lipids and proteins (e.g., malondialdehyde and protein carbonyl levels), and activity evaluation of antioxidant enzymes (e.g., glutathione peroxidase). Many studies found a cord blood profile comparable to the maternal one, with a higher level of ROS and lower antioxidant activity in the offspring of preeclamptic women, suggesting that the mother and fetus are subjected to a pro-oxidant environment (156, 174–176). Moreover, preclinical data from ewe's model support that chronic hypoxia exposure significantly increases fetal plasma urate concentration from oxidative stress (184).

Neonates of preeclamptic women with associated hemolysis, elevated liver enzymes, and low platelets (HELLP) syndrome showed higher oxidative stress up to seven days of life compared to a group with preeclampsia but without HELLP (185). Hence, fetal oxidative stress appears to be proportional to the extent of maternal endothelial dysfunction and remains even after the interruption of placental circulation. In addition, preterm infants have a lower antioxidant reserve, being more susceptible to oxidative stress injury (186). Moreover, for the same GA, lower-weight infants have less tolerance to oxidative stress, especially when comparing small for gestation age (SGA) and adequate for gestational age (AGA) (187–189).

The direct correlation between oxidative stress and early neonatal outcomes has been the subject of a few studies with inconclusive results (179, 180, 190). Conversely, late consequences of oxidative stress from dysfunctional placentation have been more extensively investigated. Lipid peroxidation levels remain higher until adolescence in infants exposed to perinatal complications, including an adverse intrauterine environment (191). Moreover, FGR rat models proved that impairment of hepatic and muscular mitochondrial oxidative processes predisposes to improper glucose metabolism, insulin resistance, and diabetes typical of these infants (192–194). Finally, preliminary data show that the maternal total antioxidant status may predict infant motor development at one year of corrected age in preeclampsia (178).

All these findings report a persistent cellular inheritance in subjects exposed to placental dysfunction, suggesting developmental programming influenced by oxidative stress (195). In this direction, studies indicate that using a ROS-targeted therapy with antioxidants may be a promising approach in perinatal medicine, especially for fetal neuroprotection (196). However, many proposed drugs are still under investigation for routine clinical use, such as vitamins, melatonin, lycopene, selenium, acetylsalicylic acid, L-ergothioneine, and mitochondria-specific drugs (197–210).

### Angiogenic factors imbalance

#### Molecular basis

The main effect of malperfusion and oxidative stress is the decreasing activity of three pivotal factors, VEGF, placental growth factor (PlGF), and transforming growth factor-β (TGF-β), which play a crucial role in placental and fetal angiogenesis (31, 211). Indeed, soluble fms-like tyrosine kinase-1 (sFlt-1), an inhibitory receptor for VEGF and PlGF, and soluble endoglin (sEng), a co-receptor for TGF-β, are over-expressed during malperfusion contributing to reducing the activity of VEGF, PlGF, and TGF-β (31). Their antiangiogenic role is exerted by binding their circulating ligands and preventing proangiogenic effects on native endothelial cell-surface receptors (31, 154). In turn, the persistent increase in sFlt-1 and the decrease in VEGF can worsen oxidative stress and impair vascular reactivity, contributing to endothelial dysfunction (212).

There are mainly two pathways that regulate angiogenic factors imbalance. First, hypoxia-inducible factors (HIFs) are oxygen-sensitive transcriptional factors that regulate the expression of the multiple vascular growth factors involved in angiogenesis (213). Specifically, HIF-1α is a subunit of HIF-1 that is rapidly inactivated and degraded in normoxia, stimulating the sFlt-1 and sEng transcription (214). In a transgenic mouse model, embryonic overexpression of HIF-1α leads to placental malformations (215). HIF-1α increases in women with preeclampsia and FGR (216, 217). Second, there is a typical deviation of the renin-angiotensin-aldosterone system with reduced plasma renin activity and the development of circulatory volume in preeclampsia. Characterization of this pathway identified a pathognomonic circulatory protein in preeclamptic women: angiotensin II type 1 agonistic receptor autoantibody (AT1-AA), inhibiting trophoblastic invasiveness by stimulating the expression of sFlt-1 and sEng (218, 219).

Over the past ten years, several studies investigated the imbalance of angiogenic factors in placental dysfunction, demonstrating increased concentrations of sFLT-1 and sENG and contextual reduction of PlGF in the plasma of women with preeclampsia and/or FGR compared to healthy controls (143, 220, 221).

#### Perinatal evidence and perspectives

Maternal plasma concentration of sFlt-1 was correlated with doppler abnormalities of the uterine and umbilical arteries on prenatal ultrasound evaluations in women with PE or FGR (222). In addition, a low plasma angiogenic index-1 (PlGF/sFLT-1 ratio) at 20–23 weeks of gestation identified patients with high maternal vascular underperfusion lesions and a higher incidence of preterm delivery (223). Moreover, the reduced placental angiogenic index-1, measured by enzyme-linked immunosorbent assays, strongly increased the risk of fetal death (224). Likewise, the increased sFlt-1/PlGF ratio correlated with fetal death, FGR, HELLP, and placental abruption in PE with high oxidative stress (225). These findings confirm that maternal endothelial dysfunction severity influences fetal and neonatal outcomes.

Angiogenic biomarkers in feto-placental circulation are less studied. However, sFLT-1 appears to increase in preeclamptic women's neonates, while data on VEGF levels are conflicting (211, 226–231). In FGR, an overall reduction in proangiogenic factors is established (226, 229). A low circulating level of insulin-like growth factor-1 (IGF-1), an anabolic hormone with proangiogenic effects, is among the documented metabolic problems of infants with a history of FGR (232).

An extensive analysis of angiogenic factors in a large cohort of infants with both endotypes of prematurity evaluated the correlation between plasma angiogenic factors at birth and placental histology (152). Two findings of this study are particularly relevant: (1) VEGF and PlGF were decreased in infants from pregnancies with maternal vascular underperfusion lesions; (2) more than 50% of severe maternal vascular underperfusion lesions were associated with FGR, while acute inflammation was associated with no or mild maternal vascular underperfusion lesions. Therefore, although preclinical and clinical data showed a reduction of proangiogenic factors even in the infectious/inflammatory endotype, a severe antiangiogenic imbalance is characteristic of chronic inflammation induced by dysfunctional placentation and should be considered in FGR (233, 234).

The consequences of angiogenic inhibition are significant. Pulmonary vasculature immaturity and poor lung growth secondary to FGR increase respiratory complications in extreme prematurity, causing high mortality in this group of infants (235). BPD development is extensively established in FGR, particularly when BPD is associated with pulmonary hypertension (11, 145, 152, 236–238). Conversely, preserving vascular growth with an sFlt-1 inhibitor or recombinant IGF-1 promotes alveolarization and sustains the architecture of the distal airspace in preclinical models (239–241). In this regard, the disruption of angiogenesis appears to be a connecting bridge between dysfunctional placentation and BPD. Additionally, the prenatal impairment of VEGF, PlGF, and IGF-1 pathways, associated with the increase in oxidative stress, mimics the pathogenesis of the first stage of ROP, suggesting a direct association between ROP and dysfunctional placentation (242, 243).

### Vasomotor regulation impairment

#### Molecular basis

Placental dysfunction is accompanied by marked alterations in vasoactive mediators, particularly gasotransmitters and endothelin-1 (ET-1) (244–246). Gasotransmitters, such as NO, hydrogen sulfide (H_2_S), and carbon monoxide (CO), are endogenously-produced, volatile molecules characterized by a high reactivity and free diffusion through cell membranes (247). Gasotransmitters exert a synergistic action of smooth muscle relaxation: NO and CO through an increase of cyclic guanosine monophosphate (cGMP) and H_2_S through K_ATP_ channels mediated hyperpolarization (247, 248).

In particular, NO is the main mediator of endothelium-dependent vasodilation, which plays a pivotal role in maintaining vascular tone (160, 249–251). It is generated *via* L-arginine oxidation by a family of NO synthase (NOS) enzymes (252). All isoforms of NOS can be endogenously inhibited by asymmetric dimethylarginine (ADMA), a methylated product of L-arginine synthesized by protein arginine methyltransferase (PRMT) and degraded by dimethylarginine dimethylaminohydrolase (DDAH) (226, 253).

Under physiological conditions, maternal gasotransmitters concentrations are significantly higher throughout pregnancy than in non-pregnant women (246, 254). They participate in trophoblast invasion and apoptosis, regulate placental blood pressure, exert antiplatelet properties in the intervillous space, enhance VEGF, and reduce sFLT-1 expression (246, 255, 256). Conversely, their reduced bioavailability, especially of NO, is one of the leading causes of abnormal placentation and endothelial dysfunction in preeclampsia (254).

ET-1 concentrations are also pathological in dysfunctional placentation (257). ET-1 is a vasoconstrictor and mitogenic peptide constitutively synthesized by endothelial cells and syncytiotrophoblasts. It can be overexpressed, mobilizing its stores in the Weibel-Palade bodies by several proinflammatory stimuli, such as hypoxia, cytokines, ROS, and shear stress (258). ET-1 induces cell proliferation and vasoconstriction binding ET-A and ET-B membrane receptors on vascular smooth muscle cells, which can persist for several hours, despite the short plasma half-life of ET-1 (257). Differently, endothelial ET-B receptors lead to NO-mediated vasodilation (259).

#### Perinatal evidence and perspectives

In general, gasotransmitters production and bioactivity are significantly reduced in preeclamptic women (246). However, there are important differences in the studies that focus on NO levels and endothelial NOS (eNOS) expression in preeclampsia (260). Moreover, nitrate and nitrite used to evaluate NO production are affected by diet and plasma clearance (244). Nevertheless, a meta-analysis showed an overall reduction in maternal serum NO level in preeclampsia and an increased risk of the condition related to genetic variations of eNOS (261). Additionally, ADMA and cGMP levels are consistently higher and lower, respectively, before and during the onset of preeclampsia, indicating decreased maternal NO bioactivity (255). These findings are supported by experimental evidence of impaired NO-mediated relaxation in vessels exposed to plasma of preeclamptic subjects and by the occurrence of FGR in pregnant rats following NOS inhibition (262–266).

Besides maternal NO levels, fetal and neonatal levels are reduced. A study by Aikio et al. found perinatal undetectable nitrate and nitrite values on airway specimens in a group of very preterm infants affected by maternal preeclampsia compared to higher values in infants with infectious/inflammatory endotype (267). Moreover, recent clinical data show higher levels of ADMA and PRMT-1 in the placenta and cord blood of FGR patients, associated with a decrease in ADMA's metabolizer DDAH-1, NO, and eNOS (226).

In addition, *ex vivo* experiments on HUVECs showed a reduction in angiogenesis with an increase in sFlt-1 after stimulation with high doses of ADMA (226). Interestingly, the link between NO bioavailability and vascular growth restriction is confirmed in cases of PROM-associated oligohydramnios. Although PROM is classified in the other endotype, it has pathophysiological overlaps with these processes. When oligohydramnios lasts more than a week, it can lead to lung growth restriction, locally simulating the lung condition of FGR (268). In the same study by Aikio et al., a group of very preterm infants affected by PROM with hypoxic respiratory failure showed undetectable perinatal nitrate and nitrite levels on airway specimens and pulmonary hypertension responsive to inhaled NO. For these reasons, the pulmonary endothelial consequences discussed in this endotype should be considered when approaching a newborn affected by long-lasting oligohydramnios (267).

In contrast to the vasodilatory gasotransmitters, ET-1 levels are increased in preeclampsia in correlation with the severity of the condition (257). Furthermore, ET-1 levels correlate with increased levels of sFLT-1 and sENG, and HIF-1 appears to regulate its synthesis (245). ET-1 physiologically contributes to high fetal pulmonary vascular resistance. Preclinical models of chronic fetal pulmonary hypertension showed a 3-fold higher ET-1, decreased ET-B receptor-mediated vasodilation, enhanced ET-A receptor-mediated vasoconstriction, and impaired angiogenesis (269, 270). Conversely, chronic intrauterine antagonism of the ET-A receptor reduces fetal pulmonary artery pressure, right ventricular hypertrophy, and distal muscularization of small pulmonary arteries, reducing pulmonary vascular resistance at birth and confirming the link between ET-1 and FGR (271).

Finally, increasing evidence indicates epigenetic pathways regulating the alterations in endogenous vasoactive mediators in the fetus (272). Histone modifications regulate eNOS expression in human umbilical artery endothelial cells (HUAEC) in patients with placental insufficiency and lead to an ET-1 increase in pulmonary vascular endothelial cells with persistent effects weeks after birth in a rat model of FGR (273, 274). In turn, the placental nitrergic system controls epigenetic mechanisms, including the function of histone deacetylase (256). Experimental data suggest that NO, ADMA, and PRMT imbalances could participate in fetal epigenetic reprogramming, providing a biological link between dysfunctional placentation and adult cardiovascular disease in FGR infants (256). This evidence may explain the long-term cardiovascular effects typical of the placental dysfunction endotype and strengthens maternal treatment as the best preventive strategy. However, research in the neonatal period is also necessary to design appropriate preventive and/or therapeutic strategies for endothelial dysfunction. Identifying epigenetic biomarkers, such as plasma microRNAs, would allow early screening and follow-up for high-risk individuals, avoiding long-term complications (275).

## Conclusions

Preterm birth syndrome impairs numerous endothelial pathways with potentially severe early and late neonatal adverse outcomes. Endotyping prematurity is a pathophysiological-based approach useful for neonatologists to predict the main endothelial features of each preterm infant, frame its early and late cardiovascular risk factors, anticipate and prevent associated hemodynamic complications, and design personalized therapeutic strategies.

Of note, the classification into endotypes is a dichotomous model and, therefore, has inherent limits to consider. First, in endothelial phenotypes, elements common to both endotypes coexist. Any cause of premature birth inevitably leads to an overlap of intrauterine inflammatory pathways, making them characteristic and not exclusive to a single endotype. On the other hand, each endotype has an inter-individual variability depending on trigger severity, timing, fetal development, and genetic background. Lastly, there is always the superimposition of postnatal “hits”, such as acidosis, hypoxia, hyperglycemia, mechanical ventilation, and sepsis, which further compromise endothelial function (60, 76, 236, 276). Hence, identifying, understanding, and quantifying the contribution of pre- and postnatal processes to endothelial dysfunction is challenging and requires an early endothelial evaluation to limit confounding factors (103). Although many fetal processes are not fully understood, the available endothelial biomarkers to perform this evaluation in NICU are numerous (76, 103, 152, 175, 260). They await further studies to validate their diagnostic and therapeutic potential. We believe that advancing endothelial characterization could be a promising way to provide patient-tailored care to the most vulnerable newborns.
